# Effects of larval diets and temperature regimes on life history traits, energy reserves and temperature tolerance of male *Aedes aegypti* (Diptera: Culicidae): optimizing rearing techniques for the sterile insect programmes

**DOI:** 10.1186/s13071-019-3830-z

**Published:** 2019-12-10

**Authors:** Hadian Iman Sasmita, Wu-Chun Tu, Lee-Jin Bong, Kok-Boon Neoh

**Affiliations:** 1 Department of Entomology, National Chung Hsing University, 145, Xingda Rd. South District, Taichung, 402 Taiwan; 2 Center for Isotopes and Radiation Application (CIRA), National Nuclear Energy Agency (BATAN), Jl. Lebak Bulus Raya No. 49, Jakarta, 12440 Indonesia

**Keywords:** Life history trait, Physiological performance, Heat tolerance, Energy reserves, Sterile insect technique

## Abstract

**Background:**

Producing high quality sterile males is vital in *Aedes aegypti* rear-and-release birth control strategies. Larval diets, rearing temperatures, and their interactions determine the accumulation rates of essential nutrients in larvae, but these factors have been understudied in relation to mass-rearing techniques for producing eminent males.

**Methods:**

We compared the effects of two larval diets, a cereal-legume-based diet (Khan’s diet) and a standard larval diet developed in the FAO/IAEA Insect Pest Control Laboratory (IAEA 2 diet). Diets were tested at selected temperatures for both larval and male adult life history traits, adult extreme temperature tolerance, and mating capacity relative to energy reserves of reared male adult *Ae. aegypti*.

**Results:**

Khan’s diet resulted in shorter immature development time at each test temperature (except for 25 °C) than an IAEA 2 diet. Larvae reared at 28 °C and 32 °C with Khan’s diet demonstrated low pupation rates (*c.*80%). We accounted for these phenomena as secondary sex ratio manipulation, because a higher proportion of male adults emerged at 28 °C and 32 °C than that for the IAEA 2 diet. In general, the pupal development time shortened as temperature increased, resulting in higher teneral energy reserves in male mosquitoes. High energy reserves allowed male mosquitoes reared with Khan’s diet to have higher adult longevity (5–6 days longer when sugar-fed and 2–3 days longer when water-fed) and tolerance of heat stress than those fed on the IAEA 2 diet. The IAEA 2 diet produced larger male mosquitoes than Khan’s diet did: mosquitoes fed on Khan’s diet were 1.03–1.05 times smaller than those fed on the IAEA 2 diet at 28 °C and 32 °C. No evidence indicated reduced mating capacity for small mosquitoes fed on Khan’s diet.

**Conclusions:**

Larvae reared at 28 °C and 32 °C with Khan’s diet were characterized by shorter immature development time compared with those fed on the IAEA 2 diet. Adult mosquitoes produced from that larval rearing condition exhibited a significant male bias, long lifespan, and better endurance against extreme temperatures relative to energy reserves. Thus, the larval diet at rearing temperature of 28 °C and 32 °C optimized rearing techniques for the sterile insect programmes. However, mating competitiveness and flight performance of adult males require further investigation.

## Background

Birth control strategies are increasingly necessary in area-wide integrated pest management (AW-IPM) to combat the spread of dengue virus transmitted by *Aedes* mosquitoes [1]. Strategies such as the classical radiation-based sterile insect technique (SIT), release of insects carrying dominant lethals, and incompatible insect technique (IIT) through *Wolbachia*-infected mosquitoes are designed to overcome the inadequacy of current control methods, which rely mostly on chemical intervention and community participation [1, 2], as well as the unavailability of licensed dengue vaccines [3]. The success of these rear-and-release strategies rely heavily on the mating competitiveness of released males; the released males must be able to outcompete wild males in seeking, courting, and mating with wild females [1]. In addition to mating competitiveness, loss of male physiological performance and poor phenotypic quality are major technical causes contributing to rear-and-release strategy failure [4, 5]. For example, excessive levels of radiation exposure in released males could reduce their effectiveness, because males suffer somatic damage. Reduced longevity and mating competitiveness are often a result of radiation-induced somatic damage [5, 6]. Similarly, studies on the effects of genetic manipulation on the fitness of mosquitoes have demonstrated that transgenic mosquitoes exhibited significantly reduced fitness, such as low survival of immature stages, reduced fecundity, and low longevity [7, 8]. In addition, in an open field release in Malaysia, transgenic mosquitoes demonstrated reduced dispersal, although no difference in longevity was observed compared with the wild-type laboratory strain [9]. The physiological performance of *Wolbachia-*infected mosquito hosts is similarly compromised. In particular, the populations of *w*MelPop-infected *Ae. aegypti* that had been released to several localities reportedly decreased in adult longevity [10, 11] and wing size during the cooler months [12]. Such limitation in quality of released *Wolbachia*-infected males remains a key challenge for IIT programs that requires addressing [13]. Released males require increased phenotypic qualities towards sexual performance to compensate for any costs that might be associated with manipulative treatment [14]. The poor physiological performances of released males may be overcome by optimizing nutrient intake that potentially results in high-quality males using mass-rearing techniques [15, 16].

Enhancing the quality of released males with adequate phenotypic quality could be achieved by optimizing larval rearing conditions. Larvae accumulate nutrients for their development and sufficient nutrient acquisition in larval stages is vital for optimal life histories during adult stage [17–20]. Larval diets for mass-rearing purposes have been thoroughly studied in several SIT mosquito-targeted species, such as *Anopheles arabiensis* [21–23], *An. stephensi* [24], *An. gambiae* (*s.s.*) [20], *Ae. albopictus* [25, 26] and *Ae. aegypti* [27–29]. In the present study, two larval diets were tested. The IAEA 2 diet is a second version of the diet developed at the FAO/IAEA, which was initially used for mass production of healthy male *Anopheles* mosquitoes for classical SIT applications (*An. arabiensis*, Damiens et al. [21]). The IAEA 2 diet contains complete essential fatty acids and amino acids required by mosquitoes. Puggioli et al. [25] reported that IAEA 2 is considered a promising diet among the three animal-derived composition diets tested (one diet developed by Centro de Agricultura Ambiente, “Giorgio Nicoli”, Crevalcore, Italy, and two diets developed by the FAO/IAEA Laboratory) for *Ae. albopictus*. In addition to cost considerations, larvae fed on IAEA 2 exhibited the shortest time to pupation and adult emergence, as well as the highest pupal production. In 2013, Khan et al. [24] developed alternative inexpensive and globally available diet ingredients: a mixture of bean, corn, wheat, chickpea, rice and bovine liver. The diet generates males of high quality in terms of their shorter larval duration and higher survival in *Anopheles* mosquitoes. In addition, cereals and legumes provide plentiful nutrients, such as carbohydrates, amino acids, polyunsaturated fatty acids (PUFAs), unsaturated fatty acids, vitamins and minerals essential for larval development, which are usually lacking in other test diets. Comparison between carbohydrate-rich diet (rodent diet (LRD) and protein-rich diet (IAEA diet) resulted in male adult *Ae. aegypti* from LRD diet being significantly larger than those IAEA diet [27].

Despite larval density [28, 30], the composition of larval diet (i.e. the ratio of carbohydrates and proteins), temperature and their interaction also determine the accumulation rate of essential nutrient in larvae. In general, temperature should allow mosquito larvae to obtain sufficient nutrients before developing to the next stage. By contrast, excessive temperatures expedite larval development and result in insufficient mass for eclosion and premature mortality [18, 31].

The study on larval physical endurance in the environment in relation to teneral energy has undergone limited examination. To date, only one larval diet study considering metabolic energy reserves as the study parameter has been performed for a single species, *An. gambiae* (*s.s.*) [20]. Sugar body content in newly emerged males is vital for teneral energy to make the first flights during the adult stage before food sources can be accessed. In addition, glucose and glycogen, as main sugars in male mosquitoes may be readily utilized and catabolized to produce ATP during starvation. Thus, we hypothesized that sugar body content may determine the life history traits of males as well as the tolerance to extreme temperatures and mating capacity to produce highly competitive male phenotypes for rear-and release strategies.

Previous studies have focused mainly on the quantities of males being produced and the mating competitiveness of released male mosquitoes, but the males’ phenotypic quality, particularly adult life history traits, have been understudied [27–29, 32]. In addition, studies of mass production of male mosquitoes have focused only on a single factor [21, 23, 24], and the interactive effects between larval diet and rearing temperature have been largely neglected. In this study, we sought the optimum environmental conditions for mass rearing of male *Ae. aegypti*. We compared two larval diets at selected temperatures by examining both larval and male adult life history traits, adult tolerance of extreme temperatures, and mating capacity relative to energy reserves of reared male adult *Ae. aegypti*.

## Methods

### Experimental colony

The *Ae. aegypti* strain used for this experiment was the Bora Bora strain. The colony was reared in the insectarium at 25.0–25.5 °C, 65–75% relative humidity, and a photoperiod of 12:12 (light:dark) h. The rearing methods used were the same as those described in Bong et al. [33]. To obtain eggs for testing, a cup layered with a filter paper was placed into a cage with blood-fed females for 2–3 days for oviposition. The eggs laid on filter paper were then air-dried for 3 days and subsequently kept inside a zip lock bag for at least 3 weeks to allow the eggs become mature before the experiments.

### Larval diet and temperatures

Two larval diets were used in this experiment. The diets were based on those of Khan et al. [24] and Damiens et al. [21], who sought the most prominent and relatively cheap diet for mass production of anopheline mosquitoes. The larval diet used by Khan et al. [24] is a carbohydrate-rich diet (hereafter referred to Khan’s diet), which contains a mixture of beans, corn, chickpeas, rice and bovine liver in equal proportions. As a refined version of the IAEA 1-larval diet developed by Damiens et al. [21], IAEA 2 was the recommended diet used in the FAO/IAEA Insect Pest Control Laboratory. The IAEA 2 diet is a protein-rich diet consisting of 25% bovine liver powder, 50% tuna meal, 12.5% brewer’s yeast, 12.5% squid powder and vitamins. Both diets were prepared in a paste form. They were stored in a refrigerator at 4 °C until use. The development and survival of mosquitoes fed either diet were observed under temperatures of 15, 20, 25, 28 and 32 °C. All experiments were conducted in environmental testing chambers (Fame/F-360DNH, Taipei, Taiwan) with a 12:12 (light:dark) h photoperiod. A total of 10 larval diet-temperature combinations were used to examine the effects of larval diets across constant different temperatures.

### Development of the immature stages and survival

Eggs were immersed in 600 ml of dechlorinated water containing 2–3 drops (1 ml) of vitamin C to induce hatching. We transferred a total of 96 L1 larvae that emerged within 12 h individually into a 24-well microtiter plate (1.5 cm in diameter and 1.5 cm in depth) filled with dechlorinated water. Prior to the daily addition of approximately 2.5 mg test diets, the water was replaced. Development time for the immature stages was recorded at 12 h intervals until adulthood was reached. The experimental design allowed examination of the effects of larval diet and temperature regimes on the development time and survival of every instar, but this method gave no gauge of the effect of larval density. The percentage of pupation and adult eclosion at each combination were generated. Sex was determined at the pupal stage. Only male mosquitoes were used for the subsequent tests.

### Pupal and wing morphometrics

Between 10 and 20 male pupae derived from each combination were immobilized by freezing (4 °C) for 5 min. The length of the cephalothorax was measured to determine pupal size [34]. The pupae were then preserved until the adult eclosion for wing measurement. The right wing (or left if the right was damaged or lost) was separated from the body under a dissecting microscope. A wing was measured from the distal edge of the alula to the end of the radius vein excluding fringe scales [35]. A digital image of the wing was captured using a camera (EI200 HD, OPTI Advanced Imaging Ltd., Taipei City, Taiwan) mounted on a stereomicroscope (Leica S8 AP0, Leica Microsystems, Heerbrugg, Switzerland). The image was analyzed using Helicon Focus version 6.7.1 Lite (Helicon Soft Ltd., Kharkiv, Ukraine).

### Longevity of the adult male

Twenty male specimens (24 hours-old) that emerged from each combination were placed in separate plastic cages (30 × 30 × 30 cm) (BugDorm 1; MegaView, Taichung, Taiwan). Two observations were made: male mosquitoes provided with 10% w/v sucrose solution and male mosquitoes provided with water only. The water-only mosquitoes tested how long a male mosquito could be sustained in an environment as if the released mosquito fails to access food sources. Three replications of observations from each combination were conducted. The mortality of sugar- and water-fed male specimens was recorded on a daily basis.

### Sugar content analyses

Five newly emerged male specimens (< 12 hours-old) from each combination were subjected to sugar content measurement to quantify teneral energy reserves. Glucose, glycogen and trehalose content were determined using methods adapted from Van Handel [36] and Leyva et al. [37]. Each male specimen was weighed using an analytical balance (Shimadzu, ATY124, Kyoto, Japan) prior to analysis. Male individual was homogenized in 0.6 ml of a 2% sodium sulfate solution (Anhydrous, Union Chemical Works Ltd., Hsinchu, Taiwan) then centrifuged at 1000×*g* for 20 min at 4 °C. Approximately 0.5–0.6 ml of the aqueous fraction was separated from its precipitate fraction and divided into two for glucose and trehalose assays, while the precipitate fraction was used for a glycogen assay.

Glucose was measured by adding 1 ml glucose assay reagent according to the manufacturer’s instructions (Sigma-Aldrich, Missouri, USA) to the 10 µl aqueous fraction and then incubating the mixture for 15 min at room temperature.

For trehalose assay, 40 µl of 1% sulfuric acid solution (98.08%, Union Chemical Works Ltd., Hsinchu, Taiwan), 40 µl of 30% potassium hydroxide solution (Union Chemical Works), and 800 µl of 0.1% anthrone reagent (ACS reagent, 97%, Sigma-Aldrich) dissolved in sulfuric acid were added to the second portion of the aqueous fraction (40 µl). Every addition process was followed by heating and cooling at 90 °C and 0 °C for 10 and 3 min, respectively.

Glycogen was determined by adding 1 ml of anthrone reagent dissolved in sulfuric acid to the precipitate fraction, followed by incubation at 90–110 °C for 17 min and sitting on ice for 3 min.

Absorbance of glucose, trehalose and glycogen were read on a spectrophotometer (Hitachi, U-2900, Tokyo, Japan) at 340, 620 and 625 nm, respectively, and the values were determined using standard curves. The standard curves were created using known concentrations ranging from 0.00 to 0.16 mg/ml of glucose and trehalose standard solution (Sigma-Aldrich) for the glucose and trehalose assays, respectively; concentrations ranging from 0.00 to 0.02 mg/ml (Sigma-Aldrich) of glycogen were used to calculate glycogen concentration.

### Heat and cold tolerance

Upper lethal temperature (ULT_50_) and lower lethal temperature (LLT_50_) that would cause 50% mortality in the adult male population from each combination were determined based on the method described by Lyons et al. [38]. Briefly, the 3–5 day-old adult male specimens were acclimatized for 1 h at room temperature prior to the analysis. Approximately 15–20 male mosquitoes were introduced into a polyethylene container (2.8 cm in diameter by 5 cm in height) and placed in the mini cooler (Major Science, MC-0203, Kyoto, Japan). The male specimens were exposed to constant temperatures of 45 °C, 44 °C, 42.5 °C, 41 °C and 40 °C for 4 h to determine ULT_50_. To determine LLT_50_, the male specimens were exposed to constant temperatures of 0 °C, − 1 °C, − 2.5 °C, − 4 °C, and − 5 °C for 4 h. The test was replicated three times for each combination.

### Mating capacity

A comparison of the mating capacity of sugar-fed male specimens from each combination was conducted. A 3-day-old virgin male specimen from each combination was introduced into the cages (30 × 30 × 30 cm) (BugDorm 1) containing ten 3–5-day-old virgin female specimens from the laboratory-reared colony for 3 days for assessment of mating activity. During these 3 days, mosquitoes were provided with 10% sugar solution. The experiment was conducted under the culture room conditions as described above. After 3 days, the mosquitoes were frozen and stored at − 20 °C until analysis. The percentage of inseminated female specimens and the number of spermathecal capsules filled with spermatozoa per female individual were determined. Four replications of sugar-fed male mosquitoes from each combination were tested.

### Data analysis

The normality of variances of each dataset was examined in advance. Any data that failed to meet the criteria of normality were log-transformed. The data expressed in percentages (larval survival, pupation, adult eclosion and female insemination) were transformed using the arcsine square-root transformation. The effects of larval diets and temperature regimes as well as their interactive effects on each parameter, were tested using univariate analysis of variance. Tukey’s honestly significant difference (HSD) *post-hoc* test was used to test significant differences in means for immature development times, survival for the immature stage (percentage of pupation and adult eclosion), pupal cephalothorax and wing lengths, sugar content, and female insemination for different temperature-larval diet combinations. Differences in the aforementioned parameters between diets were examined using Studentʼs t-test for independent samples (Additional file 1: Table S1). Pearsonʼs correlations were used to investigate the possible association between sugar content and longevity, sugar content and mating capacity, and increasing temperature and percentage of males produced. In addition, the coefficient of variation (CV, in %) of cephalothorax length was calculated to examine the degree of data dispersion around the mean. The value is indicative of the level of size homogeneity. Sex ratio and number of filled spermatheca were analyzed using nonparametric Chi-square and Kruskal-Wallis tests, respectively. Kaplan-Meier analysis was performed to estimate male adult survival function, followed by mean longevity difference comparison using the log-rank test. Heat (ULT_50_) and cold (LLT_50_) tolerance of male individuals for different combinations of larval diets and temperatures were determined using probit analysis. Overlapping confidence intervals indicate that no significant differences existed between groups. All statistical analyses were performed using SPSS analysis version 11.0 (SPSS Inc., Chicago, IL, USA) at α = 0.05.

## Results

### Development of the immature stages

**Fig. 1:**
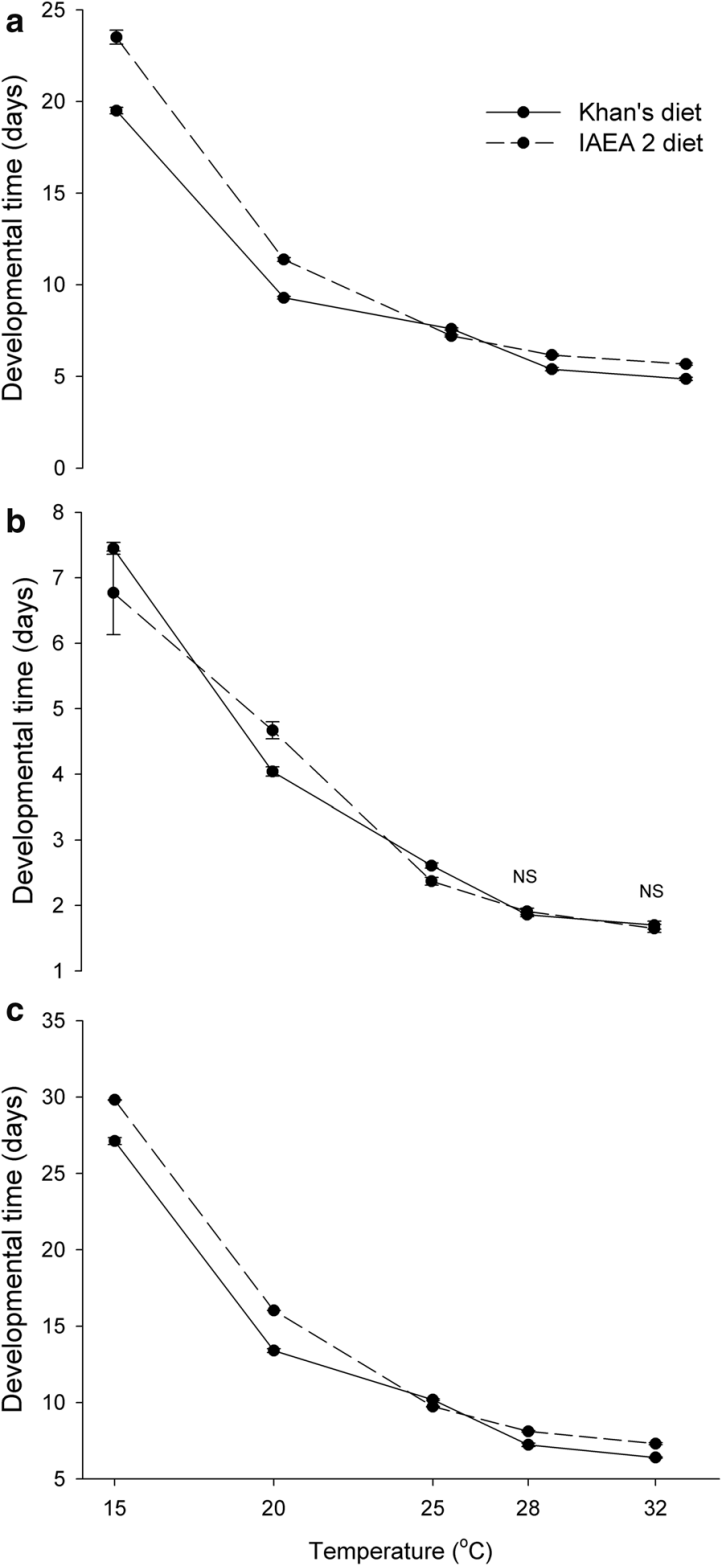
Mean development time (± SE) of the larval-stage (**a**), pupal-stage (**b**), and total immature (**c**) *Ae. aegypti* males for various combinations of larval diets and temperature regimes. NS indicates that the means for the diets are not significantly different, Studentʼs t-test for independent samples (*P* > 0.05)

The mean development times of male individuals for the larval stages L1, L2, L3 and L4, the pupal stage, and the overall development times for immature stages were significantly affected by temperature (Table 1). The mean development times for immature stages decreased with increasing temperature in both larval diets (Table 2 and Fig. 1). In particular, male larvae reared at 15 °C, 20 °C and 25 °C, regardless of diet, took 20–24 days, 9–11 days and 7–8 days, respectively, to complete the larval stage; male larvae reared at 28 °C and 32 °C required up to 5–6 days and 4–5 days, respectively, to become pupae (*P* < 0.05) (Table 2). *Post-hoc* analysis revealed that the pupation periods were significantly reduced to less than 2 days at 28 °C and 32 °C (*P* < 0.05). Overall, at the rearing temperatures of 28 °C and 32 °C, the immature stages required only 6–8 days to develop into adults.

**Table 1:** Analysis results for the variance immature life history traits and physiological performance of *Ae. aegypti* males reared with various combinations of larval diets and temperature regimes

Parameters	Factor	Type III sum of squares	*df*	Mean square	*F*-value	*P-*value
L1	Temp	14.034	4	3.508	1982.741	< 0.0001
	Diet	0.827	1	0.827	467.538	< 0.0001
	Temp * Diet	0.205	4	0.051	29.002	< 0.0001
L2	Temp	32.972	4	8.243	709.500	< 0.0001
	Diet	0.000	1	0.000	0.028	0.867
	Temp * Diet	1.199	4	0.300	25.792	< 0.0001
L3	Temp	30.814	4	7.704	703.912	< 0.0001
	Diet	0.407	1	0.407	37.172	< 0.0001
	Temp * Diet	0.147	4	0.037	3.354	0.010
L4	Temp	29.466	4	7.367	2036.100	< 0.0001
	Diet	0.253	1	0.253	69.847	< 0.0001
	Temp * Diet	0.588	4	0.147	40.629	< 0.0001
Larval stage	Temp	23.455	4	5.864	5623.814	< 0.0001
	Diet	0.365	1	0.365	350.346	< 0.0001
	Temp * Diet	0.192	4	0.048	45.942	< 0.0001
Pupal stage	Temp	16.643	4	4.161	1262.635	< 0.0001
	Diet	0.001	1	0.001	0.337	0.562
	Temp * Diet	0.144	4	0.036	10.894	< 0.0001
Total immature	Temp	14.115	4	3.529	5600.410	< 0.0001
	Diet	0.140	1	0.140	222.739	< 0.0001
	Temp * Diet	0.098	4	0.025	39.062	< 0.0001
Pupation (%)	Temp	0.759	4	0.190	5.692	0.002
	Diet	0.189	1	0.189	5.685	0.024
	Temp * Diet	0.519	4	0.130	3.889	0.012
Adult eclosion (%)	Temp	4.291	4	1.073	22.362	< 0.0001
	Diet	0.140	1	0.140	2.921	0.098
	Temp * Diet	0.671	4	0.168	3.499	0.018
Cephalothorax length	Temp	1.160	4	0.290	92.882	< 0.0001
	Diet	0.026	1	0.026	8.424	0.004
	Temp * Diet	0.043	4	0.011	3.445	0.010
Wing length	Temp	10.374	4	2.594	385.580	< 0.0001
	Diet	0.060	1	0.060	8.952	0.003
	Temp * Diet	0.132	4	0.033	4.911	0.001
Adult longevity (water-fed)	Temp	0.327	4	0.082	14.617	< 0.0001
	Diet	4.973	1	4.973	888.087	< 0.0001
	Temp * Diet	0.828	4	0.207	36.978	< 0.0001
Adult longevity (sugar-fed)	Temp	1.383	4	0.346	10.219	< 0.0001
	Diet	0.933	1	0.933	27.576	< 0.0001
	Temp * Diet	0.152	4	0.038	1.125	0.346
Glucose	Temp	1.416	4	0.354	16.999	< 0.0001
	Diet	0.217	1	0.217	10.421	0.002
	Temp * Diet	0.141	4	0.035	1.691	0.170
Glycogen	Temp	3.539	4	0.885	36.564	< 0.0001
	Diet	0.165	1	0.165	6.814	0.013
	Temp * Diet	0.814	4	0.204	8.410	< 0.0001
Trehalose	Temp	0.154	4	0.038	0.976	0.431
	Diet	0.732	1	0.732	18.606	< 0.0001
	Temp * Diet	0.160	4	0.040	1.015	0.411
Female insemination	Temp	0.010	4	0.003	0.270	0.895
	Diet	0.016	1	0.016	1.671	0.206
	Temp * Diet	0.021	4	0.005	0.544	0.705

*Abbreviation*: Temp, temperature

*Notes*: *P* < 0.05 indicative of significant effect of factors

**Table 2:** Mean (± SE) developmental time and longevity of *Ae. aegypti* males reared with various combinations of larval diets and temperature regimes

Larval diets	T (°C)	Development time (days ± SE)							Longevity (days ± SE)	
		L1	L2	L3	L4	Larval stage	Pupal stage	Total immature	Water	Sugar
Khan’s	15	5.32 ± 0.08^a^ (*n* = 48)	3.01 ± 0.06^a^ (*n* = 48)	3.37 ± 0.06^a^ (*n* = 48)	7.79 ± 0.12^a^ (*n* = 48)	19.50 ± 0.18^a^ (*n* = 48)	7.45 ± 0.09^a^ (*n* = 21)	27.11 ± 0.23^a^ (*n* = 21)	8.83 ± 0.16^a^	22.52 ± 3.12^a^
	20	2.99 ± 0.04^b^ (*n* = 53)	1.52 ± 0.05^b^ (*n* = 53)	1.62 ± 0.06^b^ (*n* = 53	3.15 ± 0.03^b^ (*n* = 53)	9.29 ± 0.07^b^ (*n* = 53)	4.04 ± 0.07^b^ (*n* = 43)	13.40 ± 0.13^b^ (*n* = 43)	6.73 ± 0.08^b^	36.63 ± 2.97^b^
	25	2.53 ± 0.01^c^ (*n* = 54)	1.17 ± 0.03^c^ (*n* = 54)	1.08 ± 0.03^b^ (*n* = 53)	2.77 ± 0.04^c^ (*n* = 54)	7.59 ± 0.05^c^ (*n* = 54)	2.61 ± 0.04^c^ (*n* = 36)	10.18 ± 0.06^c^ (*n* = 36)	6.91 ± 0.10b	22.90 ± 2.29a
	28	1.88 ± 0.03^d^ (*n* = 51)	0.68 ± 0.03^d^ (*n* = 51)	1.00 ± 0.03^c^ (*n* = 51)	1.82 ± 0.04^d^ (*n* = 51)	5.39 ± 0.08^d^ (*n* = 51)	1.86 ± 0.03^d^ (*n* = 40)	7.23 ± 0.11^d^ (*n* = 40)	7.84 ± 0.12^c^	37.23 ± 4.07^b^
	32	1.55 ± 0.03^e^ (*n* = 44)	0.95 ± 0.02^e^ (*n* = 43)	0.70 ± 0.04^d^ (*n* = 44)	1.63 ± 0.06^e^ (*n* = 43)	4.86 ± 0.08^e^ (*n* = 44)	1.70 ± 0.06^e^ (*n* = 31)	6.40 ± 0.04^e^ (*n* = 31)	8.63 ± 0.13^a^	37.38 ± 4.06^b^
IAEA 2	15	6.09 ± 0.06^a^ (*n* = 46)	3.96 ± 0.09^a^ (*n* = 46)	4.29 ± 0.08^a^ (*n* = 46)	9.17 ± 0.26^a^ (*n* = 46)	23.51 ± 0.38^a^ (*n* = 46)	6.77 ± 0.19^a^ (*n* = 26)	29.81 ± 0.64^a^ (*n* = 26)	4.56 ± 0.19^a^	16.23 ± 1.79^a^
	20	3.32 ± 0.05^b^ (*n* = 48)	1.92 ± 0.04^b^ (*n* = 48)	1.93 ± 0.04^b^ (*n* = 48)	3.93 ± 0.26^b^ (*n* = 47)	11.37 ± 0.11^b^ (*n* = 48)	4.67 ± 0.06^b^ (*n* = 47)	16.03 ± 0.13^b^ (*n* = 47)	5.03 ± 0.10^b^	25.67 ± 2.82^bc^
	25	2.79 ± 0.03^c^ (*n* = 44)	0.89 ± 0.03^c^ (*n* = 44)	1.22 ± 0.03^c^ (*n* = 44)	2.28 ± 0.03^c^ (*n* = 44)	7.20 ± 0.04^c^ (*n* = 44)	2.37 ± 0.04^c^ (*n* = 28)	9.73 ± 0.06^c^ (*n* = 28)	5.71 ± 0.09^c^	18.90 ± 1.62^ab^
	28	2.50 ± 0.01^d^ (*n* = 54)	0.57 ± 0.02^d^ (*n* = 53)	1.03 ± 0.01^d^ (*n* = 54)	2.03 ± 0.05^d^ (*n* = 54)	6.17 ± 0.06^d^ (*n* = 53)	1.91 ± 0.03^d^ (*n* = 35)	8.11 ± 0.05^d^ (*n* = 35)	5.10 ± 0.08^b^	20.90 ± 0.90^abc^
	32	2.11 ± 0.02^e^ (*n* = 56)	0.87 ± 0.02^c^ (*n* = 56)	0.75±0.04^e^ (*n* = 56)	1.91 ± 0.03^d^ (*n* = 56)	5.67 ± 0.05^e^ (*n* = 56)	1.65 ± 0.03^e^ (*n* = 36)	7.31 ± 0.06^e^ (*n* = 36)	5.50 ± 0.17^bc^	28.43 ± 1.84^c^

*Notes*: Mean values followed by the same letter in the same column within each larval diet are not significantly different, Tukey’s HSD text (*P* < 0.05)

*Abbreviations*: n, sample size; T, temperature

Larval diets significantly affected the mean development times of male individuals for the larval stages L1, L3 and L4 (except for L2), and the immature stages in general (Table 1). The larvae fed on Khan’s diet developed 1–2 days faster at each test temperature except for 25 °C compared with those fed on the IAEA 2 diet (Fig. 1). In addition, the effect of diet on the pupal stage depended on temperature (Table 1). In particular, t-tests revealed that the mean development time for the pupal stage differ significantly for the two larval diets tested at larval rearing temperatures of 15 °C, 20 °C and 25 °C (*P* < 0.05) but not at 28 °C and 32 °C (*P* > 0.05) (Fig. 1, Additional file: Table S1).

### Survival of the immature stages

**Fig. 2:**
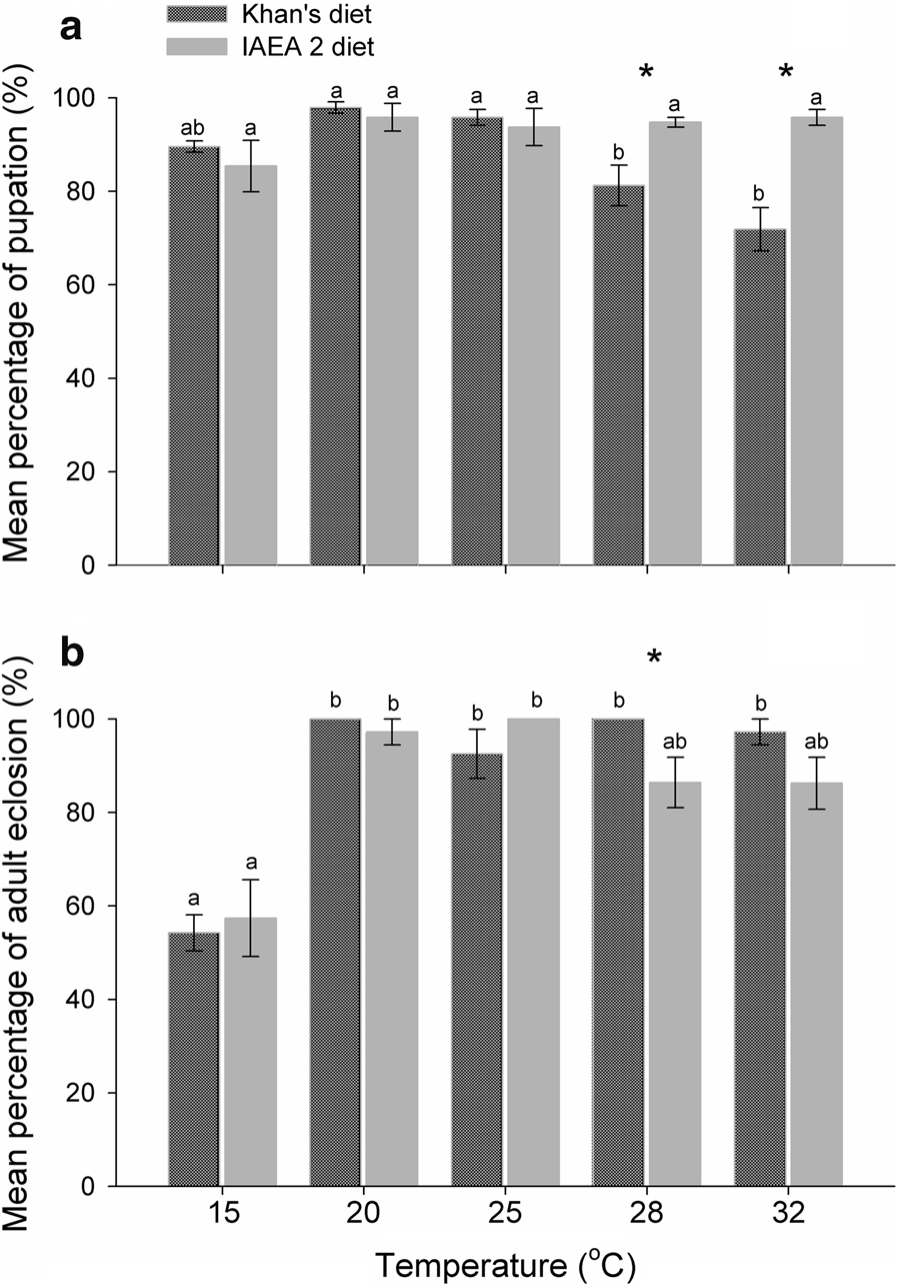
Mean percentage of pupation (**a**) and adult eclosion (**b**) for *Ae. aegypti* reared using various combinations of larval diets and temperature regimes. Bars sharing the same letter within the same diet are not significantly different, Tukey’s HSD test, *P* ≥ 0.05. (*) indicates that the means for the diets are significantly different, Studentʼs t-test for independent samples, *P* < 0.05. Error bars represent the standard error

The percentage of pupation was significantly affected by the temperature and larval diet tested (Table 1). *Post-hoc* analysis revealed that the percentage of pupation for larvae fed on Khan’s diet at 28 °C (81.25 ± 4.33%) and 32 °C (71.87 ± 4.62%) were significantly lower (*F*_(4, 15)_ = 12.661, *P* < 0.001) than those for larvae fed on the same diet at other test temperatures (15 °C: 89.58 ± 1.20 %; 20 °C: 97.92 ± 1.20 %; 25 °C: 95.83 ± 1.70%). The percentage of pupation was also recorded to be significantly lower than that for the IAEA 2 diet (up to 95% of pupation) (*P* < 0.05) (Fig. 2a and Additional file: Table S1).

The percentage of adult eclosion was significantly affected by temperature (Table 1). In particular, the mean percentage of adult eclosion at 15 °C was the lowest among temperature regimes (Khan’s diet: 54.23 ± 3.87%; IAEA 2 diet: 57.37 ± 8.22%). Percentage of adult eclosion was high when male pupae were kept at 20 °C (Khan’s diet: 100%; IAEA 2 diet: 97.22 ± 5.56%) and 25 °C (Khan’s diet: 92.52 ± 1.04%; IAEA 2 diet: 100%) (Fig. 2b). The main effect of larval diet on percentage of male eclosion was statistically non-significant, but the effect depended on temperature (Table 1). For example, the percentage of adult eclosion significantly increased up to 100% for Khan’s diet compared with those for the IAEA 2 diet, for which rates reached approximately 86%, when the larval mosquitoes were reared at 28 °C (Fig. 2b and Additional file: Table S1).

### Pupal and wing morphometrics

**Fig. 3:**
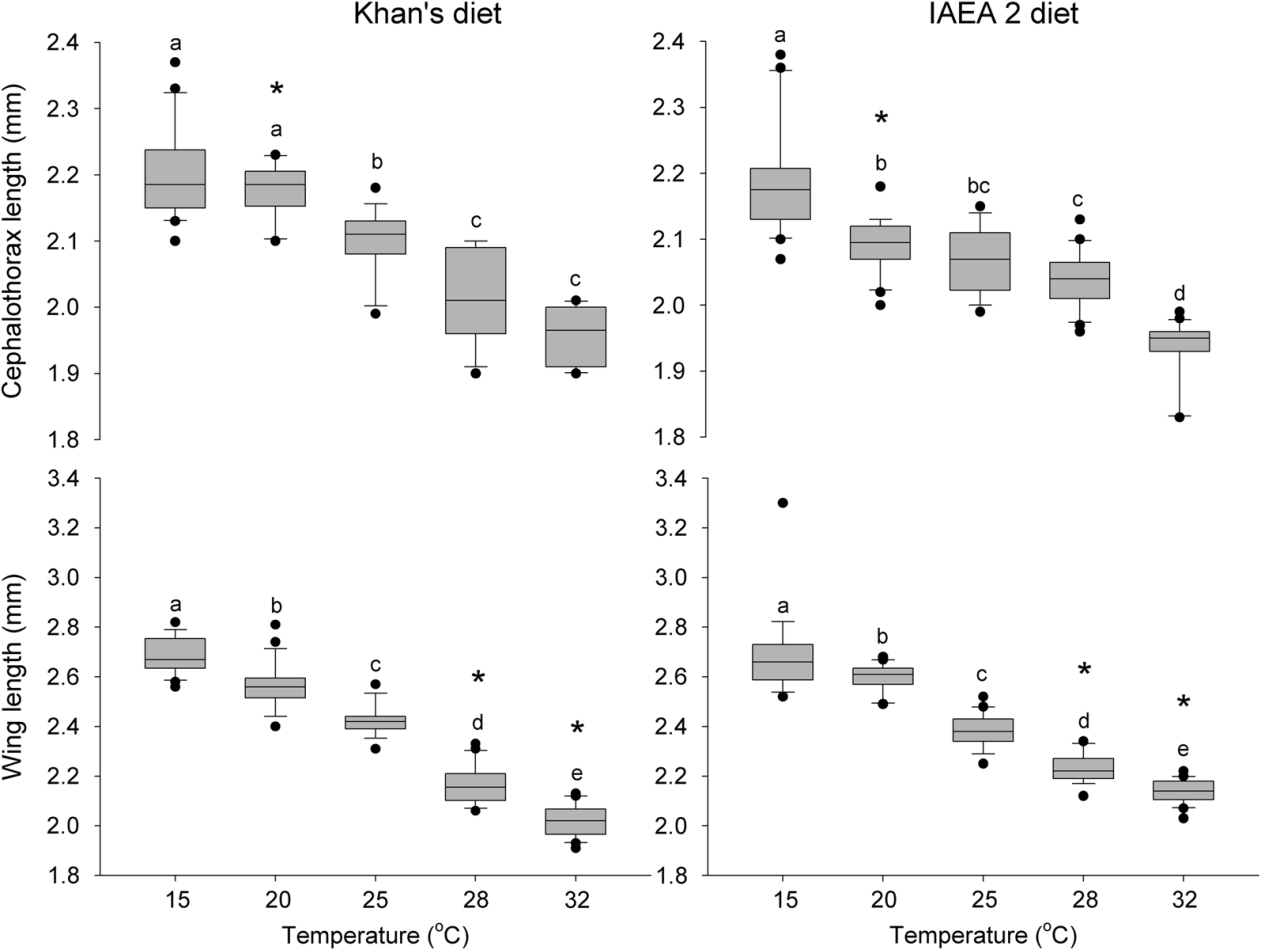
Box plots of cephalothorax and wing lengths for *Ae. aegypti* males reared using various combinations of larval diets and temperature regimes. Box plots with the same letter exhibit no significant differences among test temperatures, Tukey’s HSD test, *P* ≥ 0.05. Horizontal bars indicate median values, boxes show the lower and upper quartiles of the datasets, and outliers are represented by solid circles. (*) indicates that the means for the diets are significantly different, Studentʼs t-test for independent samples, *P* < 0.05. Error bar represent the standard error

The cephalothorax and wing lengths of male specimens were significantly influenced by temperature, larval diets, and the interaction between temperature and larval diet (Table 1). In general, the cephalothorax of male pupae reared at 28 °C and 32 °C was 1.02–1.15 times smaller than that of larvae reared at 25 °C or lower. CVs of the cephalothorax length were inconsistent for the increased temperatures tested under Khan’s diet and the IAEA 2 diet. For example, CVs of the cephalothorax length for pupae reared under Khan’s diet were 3.03%, 1.83%, 2.36%, 3.24% and 2.19% at 15 °C, 20 °C, 25 °C, 28 °C and 32 °C, respectively, and under the IAEA 2 diet were 3.78%, 1.95%, 2.52%, 2.03% and 2.63%. Similarly, the wing sizes of male mosquitoes derived from larvae reared at 28 °C and 32 °C were 1.06–1.32 times smaller than those of male mosquitoes derived from larvae reared at other temperatures. In addition, the results demonstrated that male larval mosquitoes reared at 28 °C and 32 °C with Khan’s diet possessed significantly shorter wings (2.16 ± 0.01 mm and 2.02 ± 0.01 mm, respectively) than those reared at 28 °C and 32 °C (2.23 ± 0.01 mm and 2.13 ± 0.01 mm, respectively) with the IAEA 2 diet (Fig. 3).

### Sex ratio

There was no significant correlation between increasing temperature and percentage of males (*r*_(40)_ = 0.247, *P* = 0.125). However, of all rearing conditions with different larval diets and temperatures, the pupal sex ratio of larvae fed on Khan’s diet under 28 °C and 32 °C was most significantly male-biased (Table 3).

**Table 3:** Adult sex ratio of *Ae. aegypti* reared with various combinations of larval diets and temperature regimes

Diet	T (° C)	Sex		Sex ratio (M:F)	Statistical output
		Male	Female		
Khan’s	15	48	37	1.297	*χ*^2^ = 1.424, *df* = 1, *P* = 0.233
	20	53	40	1.325	*χ*^2^ = 1.817, *df* = 1, *P* = 0.178
	25	54	36	1.500	*χ*^2^ = 3.600, *df* = 1, *P* = 0.058
	28	48	30	1.600	*χ*^2^ = 4.154, *df* = 1, *P* = 0.042
	32	44	22	2.000	*χ*^2^ = 7.333, *df* = 1, *P* = 0.007
IAEA 2	15	46	35	1.314	*χ*^2^ = 1.494, *df* = 1, *P* = 0.222
	20	48	46	1.043	*χ*^2^ = 0.043, *df* = 1, *P* = 0.837
	25	44	46	0.956	*χ*^2^ = 0.044, *df* = 1, *P* = 0.833
	28	54	40	1.350	*χ*^2^ = 2.085, *df* = 1, *P* = 0.149
	32	56	40	1.400	*χ*^2^ = 2.667, *df* = 1, *P* = 0.102

*Abbreviations*: F, female; M, male; T, temperature

### Longevity of adult male mosquitoes

**Fig. 4:**
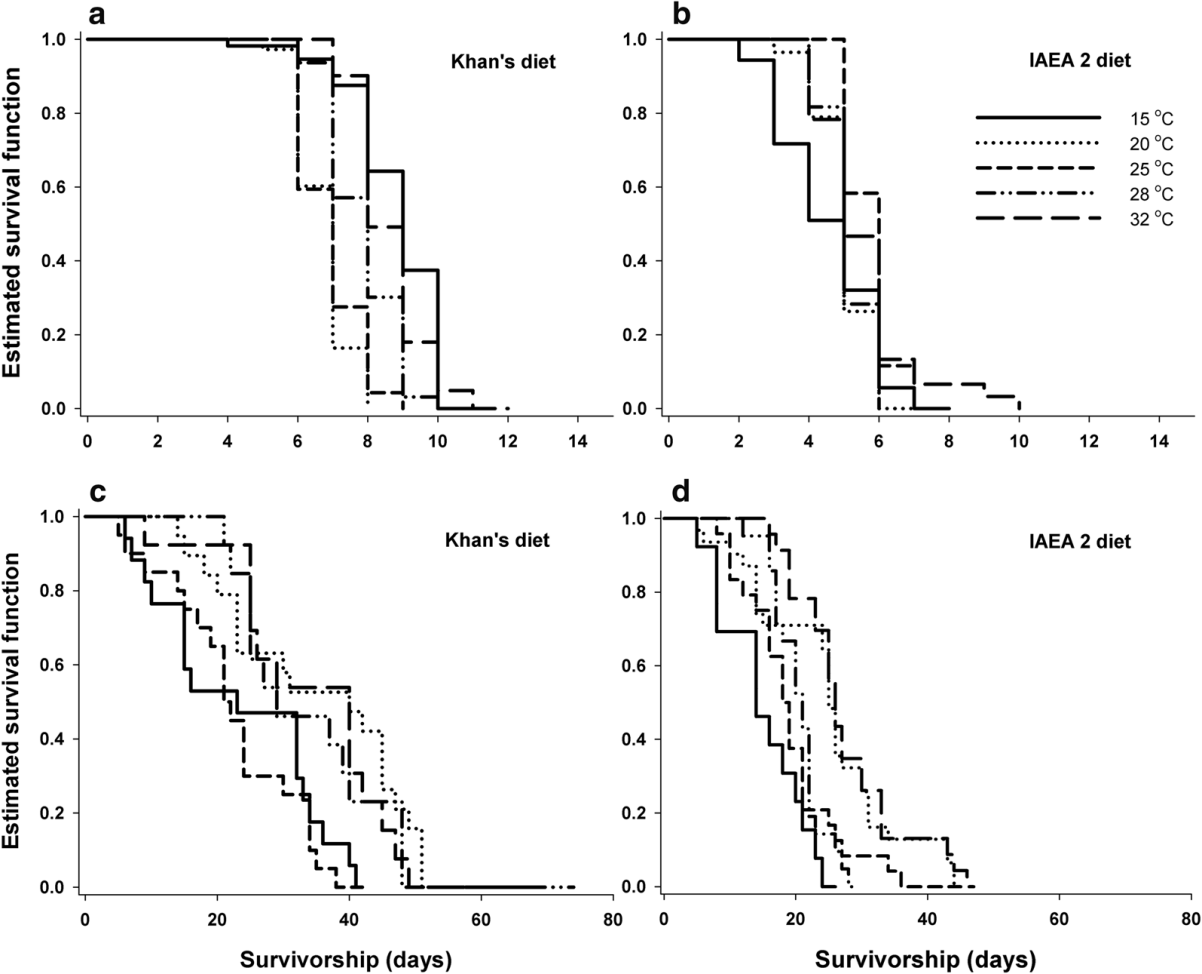
Survival patterns of water-fed (**a**, **b**) and sugar-fed (**c**, **d**) *Ae. aegypti* males for various combinations of larval diets and temperature regimes

Male adult longevity was significantly influenced by temperature and larval diet (Table 1). A statistically significant interaction indicated that the effects of temperature on water-fed male specimens longevity depended on the larval diet, but a non-significant interaction was observed for sugar-fed male specimens (Table 1). For sugar-fed male specimens, mean longevity of adults derived from larvae fed on Khan’s and IAEA 2 diet was significantly longer when larvae were reared at high temperatures (log-rank test: Khan’s diet: *χ*^2^ = 24.838, *df* = 4, *P* < 0.001; IAEA 2 diet: *χ*^2^ = 28.141, *df* = 4, *P* < 0.001). For instance, mean longevity of male specimens derived from larvae reared at 28 °C and 32 °C using IAEA 2 diet was 28.43 ± 1.84 days, which was significantly longer than those reared at 15 °C (16.23 ± 1.79 days) (Table 2). More than 90% of sugar-fed male adults derived from Khan’s diet at 28 °C and 32 °C survived for up to 22 days; the corresponding value for the IAEA 2 diet was up to 18 days (Fig. 4). By contrast, although the longevities were significantly different across temperatures tested (log-rank test: Khan’s diet: *χ*^2^ = 164.923, *df* = 4, *P* < 0.001; IAEA 2 diet: *χ*^2^ = 29.669, *df* = 4, *P* < 0.001), the mean longevity of water-fed male adult mosquitoes did not consistently increase with the rearing temperature. Approximately 50% of the male adult population derived from larvae fed on Khan’s diet survived 22–42 days compared with male adult population derived from larvae fed on the IAEA 2 diet, which survived for 16–26 days regardless of temperatures. Even with no sugar supply, overall, at least 80% of the male population derived from Khan’s diet survived for 6–8 days, longer than male specimens derived from larvae fed on the IAEA 2 diet, for which the corresponding value was 3–5 days regardless of temperature (Fig. 4).

### Sugar content analyses

Temperature, larval diet, and interaction between temperature and larval diet exerted various influences on glucose, glycogen and trehalose levels (Table 1). Glucose levels in adult males were affected by temperature and larval diet. Temperature, diet, and the interaction between the two significantly affected glycogen levels (Table 1). In general, the glucose and glycogen levels increased along with temperature, though glucose levels at 15°C–28°C were not significantly different for both larval diets (Table 4). *Post-hoc* analysis revealed that significant differences in glucose levels occurred when the larvae were reared at 32 °C on Khan’s diet (27.96 ± 3.82 µg/mg insect weight; *P* < 0.05); this value was 2.18–3.91 times higher than those for the other lower temperatures tested. By contrast, the larvae reared at 32 °C on the IAEA 2 diet showed glucose levels of 12.82 ± 2.01 µg/mg insect weight, i.e. 1.31–2.46 times higher than those for the other lower temperatures tested. The highest glycogen levels were registered for individuals fed on Khan’s diet (29.97 ± 7.50 µg/mg insect weight) and on the IAEA 2 diet (8.54 ± 0.91 µg/mg insect weight) at 32 °C. In particular, the values for Khan’s and IAEA 2 diets were, respectively, 3.45–10.93 and 1.52–3.89 times higher than those for the same diets tested at lower temperatures. Trehalose levels were not significantly different across temperatures; only larval diet affected trehalose levels (Tables 1, 4). In particular, the mean trehalose levels of adult male individuals fed on the IAEA 2 diet during the larval stage ranged from 11.33 to 18.95 µg/mg insect weight; the corresponding values for those fed on Khan’s diet ranged from 7.53 to 12.38 µg/mg insect weight (Table 4). Pearsonʼs correlation analysis revealed that sugar content and water-fed male specimens were not significantly correlated (glucose *r*_(10)_ = 0.519, *P* = 0,124; glycogen *r*_(10)_ = 0.489, *P* = 0.152; trehalose *r*_(10)_ = − 0.588, *P* = 0.074).

**Table 4:** Energy metabolic reserves and temperature tolerance of *Ae. aegypti* males reared with various combinations of larval diets and temperature regimes

Larval diet	T (°C)	Sugar content (µg/mg insect weight)			Temperature tolerance (°C)	
		Glucose	Glycogen	Trehalose	ULT_50_	LLT_50_
Khan’s	15	7.14 ± 0.65^a^	3.46 ± 0.31^a^	7.53 ± 0.63^a^	30.42 (–)	n.a.
	20	7.90 ± 1.50^a^	3.73 ± 0.68^a^	8.75 ± 1.44^a^	36.44 (28.72–38.65)	− 1.43 (− 1.66– − 1.26)
	25	8.87 ± 0.74^a^	2.74 ± 0.19^a^	8.27 ± 3.06^a^	41.74 (32.99–44.08)	− 1.11 (− 2.13– − 1.00)
	28	12.78 ± 2.36^a^	8.68 ± 1.18^b^	9.17 ± 3.42^a^	42.06 (40.80–43.09)	− 1.80 (− 3.23–0.29)
	32	27.96 ± 3.82^b^	29.97 ± 7.50^c^	12.38 ± 1.82^a^	42.79 (42.42–43.17)	− 1.21 (− 1.52– − 0.87)
IAEA 2	15	5.21 ± 0.40^a^	2.19 ± 0.24^a^	11.33 ± 1.38^a^	na	na
	20	6.48 ± 0.38^ab^	3.11 ± 0.59^ab^	14.11 ± 0.68^a^	40.01 (39.701-40.321)	− 2.10 (-2.25– − 1.96)
	25	9.74 ± 3.61^ab^	6.88 ± 1.32^c^	18.95 ± 2.99^a^	41.41 (39.62–42.55)	− 2.32 (− 2.46– − 2.19)
	28	9.56 ± 2.70^ab^	5.60 ± 0.47^bc^	15.00 ± 2.08^a^	42.2 (41.28–43.06)	− 1.05 (–)
	32	12.82 ± 2.01^b^	8.54 ± 0.91^c^	16.39 ± 4.58^a^	42.93 (41.23–45.82)	− 0.29 (–)

*Abbreviations*: na, not applicable due to mortality of > 90% for each test temperature; (–), fiducial limit not generated due to high mortality occurring when temperature decreased; T, temperature

*Notes*: Mean values followed by the same letter in the same column within each larval diet are not significantly different, Tukey’s (HSD) (*P* < 0.05). Temperature tolerance was analyzed using probit analysis

### Heat and cold tolerance

In general, male larval mosquitoes reared at high temperatures with Khan’s and IAEA 2 diets tended to be more tolerant of high temperatures during the adult stage, although the confidence intervals overlapped greatly (Table 4). The results demonstrated that the temperature required to kill 50% of the male adult population ranged from 41 °C to 43 °C when the larvae were reared between 25 °C and 32 °C; heat tolerance of male adult mosquitoes was less than 40 °C when the larvae were reared between 15 °C and 20 °C. For cold tolerance, LLT_50_ of male adult specimens fed on Khan’s diet during the larval stage consistently ranged from − 1.11 °C to − 1.80 °C across the test temperatures. By contrast, LLT_50_ of male adult individuals fed on the IAEA 2 diet was as low as − 2.32 °C (25 °C of the rearing temperature) to − 0.29 °C (32 °C of the rearing temperature) (Table 4).

### Mating capacity

**Fig. 5:**
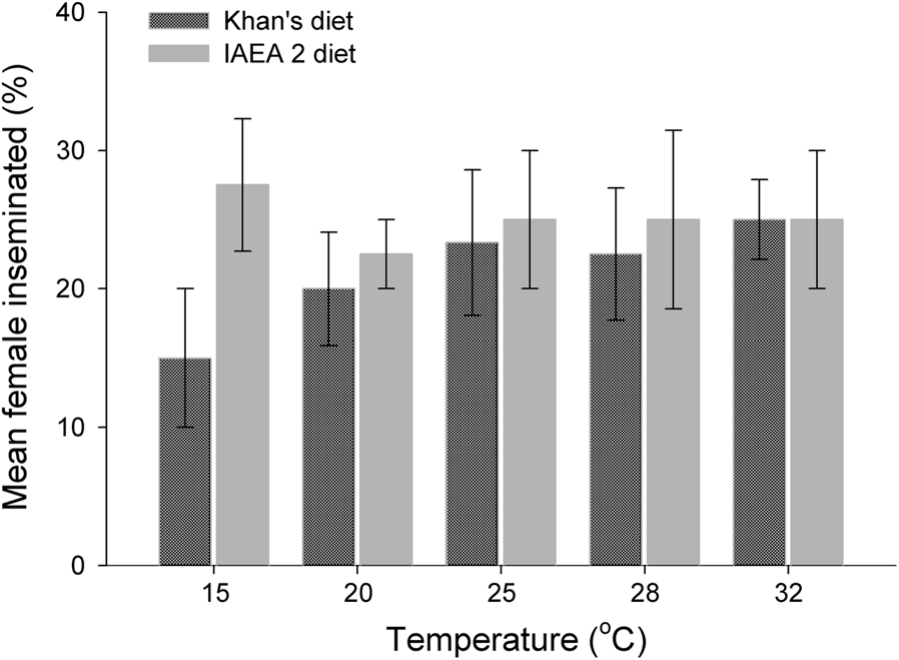
Mean percentage (± SE) of inseminated females by a single *Ae. aegypti* male reared using various combinations of larval diets and temperature regimes*. Post-hoc* tests revealed no significant difference for both diets across temperatures tested (*P* > 0.05)

The results demonstrated no significant differences for the mean percentages of female individuals inseminated by male individuals reared at different temperatures and with different larval diets (Table 1). In general, 15.0–27.5% of female individuals for each replication were inseminated by a single male individual (Fig. 5). Most male individuals from all combination treatments were able to fill two spermathecae, with the next greatest number able to fill one spermatheca and then three spermathecae (Khan’s diet: *χ*^2^ = 17.07, *df* = 2, *P* < 0.001; IAEA 2 diet: *χ*^2^ = 16.89, *df* = 2, *P* < 0.001). For both larval diets, the ability of male individuals to fill three spermathecae differed significantly for larvae reared at 20 °C (Table 5) compared with those reared at other test temperatures; more male specimens derived at 28 °C and 32 °C filled two spermathecae (this also applied for larvae reared at 15 °C with the IAEA 2 diet) (Table 5). The correlation between the number of inseminated females and mean sugar contents was not significant (glucose *r*_(10)_ = 0.221, *P* = 0,539; glycogen *r*_(10)_ = 0.210, *P* = 0.561; trehalose *r*_(10)_ = 0.631, *P* = 0.050).

**Table 5:** Numbers of females that contained spermathecal capsule(s) filled by *Ae. aegypti* males reared with various combinations of larval diets and temperature regimes

Diet	T (° C)	No. of inseminated females (*n* = 40 for each temperature tested)	No. of females that contained spermathecal capsule(s) filled		
			1 capsule	2 capsules	3 capsules
Khan’s	15	6	2	4	0
	20	9	2	4	3
	25	9	4	4	1
	28	9	2	7	0
	32	10	3	7	0
Chi-square test			*χ*^2^ = 4.018, *df* = 4, *P* = 0.404	*χ*^2^ = 8.182, *df* = 4, *P* = 0.085	*χ*^2^ = 13.247, *df* = 4, *P* = 0.010
IAEA 2	15	11	2	9	0
	20	9	2	4	3
	25	10	4	4	2
	28	10	2	7	1
	32	12	3	7	2
Chi-square test			*χ*^2^ = 7.387, *df* = 4, *P* = 0.117	*χ*^2^ = 17.516, *df* = 4, *P* = 0.002	*χ*^2^ = 9.997, *df* = 4, *P* = 0.040

*Abbreviation*: T, temperature

## Discussion

Mass production of *Ae. aegypti* requires optimal temperature and balanced nutrition to promote fast and homogenous larval development, and high phenotypic quality male mosquitoes. Both temperature and nutrition affect the performance of sterile males in the field, e.g. flight capacity, resilience in the environment, high endurance against climatic adversity, and competitiveness with wild male individuals to mate, which is important for classical SIT. Male phenotypic qualities are determined as early as the larval stages, because biosynthesis of energy reserves is significant during fourth-instar larval stage [39]. Our results elucidated that both temperatures and larval diets interactively influence immature development, sex allocation, male adult life parameters, teneral energy and heat tolerance.

### Immature development

The effect of temperature on mosquito immature development is significant. The increasing temperature elevates mosquito body temperature, leading to an increase in metabolism and respiration rate. The elevated temperatures may also affect the nervous and endocrine systems, which are responsible for insect metamorphosis [40]. Thus, the developmental rate of immature stages is temperature-dependent as increasing of temperature within particular range (14–38 °C) is negatively correlated with developmental time and body size [28, 41–47]. A minimum temperature threshold for larval development of *Ae. aegypti* is set at 8.3 °C [43]. In the present study, although larval survival at 15 °C was as high as at other temperatures, significant pupal mortality was observed (54–57%, compare Fig. 2a and b). At low temperatures, mosquitoes took significantly longer to forage for food, potentially resulting in increased resource and labor costs, making 15 °C unfavorable for mass rearing. Raising temperatures to shorten immature development time is favorable and cost-effective for male mass production [15, 25, 48], but our study demonstrated that rapid larval development contributed life history trait trade-offs.

In this study, high percentage of pupation and adult eclosion were observed when the larvae were maintained at 20–32 °C (Fig. 2). The result is in line with findings by Tun-Lin et al. [43] and Farjana et al. [49] in that the optimum temperature range for survival of larvae fed on standardized rodent diet mixed with animal liver powder as well as with yeast extract powder was between 20 °C and 30 °C; survival of the immature stages was significantly reduced at both 15 °C and 35 °C. Our study demonstrated that the percentage of pupation was interactively affected by temperature and diet type. At 28–32 °C, high temperatures accelerated the development of mosquitoes compared with the other test temperatures (15–25 °C). However, the larvae at each larval stage must achieve a minimum larval mass before progressing to the next stage [18]. In particular, larvae are required to accumulate minimum threshold of proteins and lipids, when reaching the final fourth-instar, for developing to the next stage [39]. Otherwise, the poor nutritional condition reduces energy reserve accumulation in *Ae. aegypti* and prompts premature mortality [39]. Even, fast development at high temperatures [18] under low quality nutrition [32] may cause insufficient nutrient intake, and these conditions are detrimental to mosquito larval development. The rearing combinations at 28–32 °C may therefore explain why the low percentage of pupation (70–80%) occurred when larvae were fed on Khan’s diet, which probably has a lower protein content than the IAEA 2 diet does (pupation of 97–100%) (Fig. 2). Although the differences were not statistically significant, pupae derived from larvae fed on Khan’s diet exhibited higher percentage of adult eclosion than those derived from larvae fed on the IAEA 2 diet.

### Sex ratio

The production of the cheaper sex (male) is favored under poor environmental conditions to increase the population number by increasing the mating probability in a population [50]. Gunathilaka et al. [29] reported that in *Ae. aegypti*, a relatively high proportion of male individuals was produced from the larvae reared with less food (10–20 ml), whereas the sex ratio was female-skewed when the larvae were fed with more food (40–50 ml). For *Ae. albopictus*, Puggioli et al. [26] reported that a high percentage of pupal male specimens was obtained when the larvae were reared at the lowest diet concentrations in their experiment setup. The sex ratios for the IAEA 2 diet at the four temperatures tested (except for 25 °C) demonstrated a slight male bias, but this was non-significant. Our results agreed with those from the study by Bond et al. [27], who tested the IAEA 2 and LRD diets on *Ae. aegypti* under two photo phase regimes, in which the overall adult sex ratio exhibited a slight male bias using the IAEA 2 diet, ranging from 1.17:1 to 1.23:1 (male:female), compared with using the LRD diet (0.98:1 to 1.02:1). Our results indicate that the sex ratio difference could not be explained exclusively by the effect of larval diet, but temperature should also be factored in. Significant male bias was observed for pupal male individuals when *Ae. aegypti* larvae were fed on Khan’s diet at 28 °C and 32 °C but not at 25 °C and below. By contrast, more female individuals were produced at high temperatures of 28 °C and above with a high-nutrition diet [45, 51]. This finding is likely explained by the notion that mosquito larvae may exhibit considerable differences in the survival of each sex, causing male skew, as evidenced by the low pupation rate with Khan’s diet at 28 °C and 32 °C. In *Ae. aegypti*, the critical weight of male fourth-instar larvae for triggering the molting process at 32 °C is 1.66 mg, which is lower than the corresponding weight for female individuals (1.99 mg) [18]. Male larvae must accumulate a relatively low level of energy reserves compared with female larvae before pupation to ensure survival at the next stage [18]. Thus, a likelihood exists that a high proportion of female larvae was killed before becoming pupae at high temperatures when fed relatively nutritionally poor diets owing to insufficient mass and energy reserves for pupation.

### Mosquito size

Pupal cephalothorax length was inversely related to temperature (Fig. 4). The pupal size also tended, although less consistently, to exhibit low CVs for both test diets and across temperatures. Homogeneous size of reared pupae and synchronicity of pupation onset are critical to ensure the efficiency of mechanical sex separation procedures during the pupal stages [26, 27]. Along with the considerable differential survival of sexes causing a male skew in certain circumstances, this form of sexual selection for survival may further increase the efficiency of male mosquito mass production.

More nutrients are usually acquired over extended periods of larval development [32], and optimal nutrition acquisition during larval development produces large mosquitoes [27, 52–55]. Bond et al. [27] demonstrated that the LRD diet, containing 10% more protein than the IAEA diet, produced larger adult male and female specimens. In our study, male mosquitoes were larger when fed on the IAEA 2 diet than when they were fed on Khan’s diet. The difference was, however, not significant unless the larvae were reared at high temperatures. For instance, Khan’s diet produced 1.03–1.05 times smaller male mosquitoes at 28 °C and 32 °C than the IAEA 2 diet (Fig. 3). Larger mosquitoes are favored, as these are more likely to survive [56, 57] and potentially produce more sperm [58, 59]. However, our study argues against this.

### Adult longevity and energy reserves

In this study, we observed male longevity in two different situations: male specimens could access either sugar solution or water only. This study demonstrated that, although the male mosquitoes fed on Khan’s diet were generally small, the adults exhibited higher longevity than those fed on the IAEA 2 diet in both the sugar- and water-fed experiments (Table 2). Rather than body size [56], the lifespan increase may have been associated with the high teneral energy reserves in male mosquitoes. Male mosquitoes fed on Khan’s diet exhibited glucose and glycogen levels as high as 27.96 μg/mg insect weight and 29.97 μg/mg insect weight, respectively, values at least 2–3-fold higher than for those reared on IAEA 2 diets (glucose: 12.82 μg/mg insect weight; glycogen: 8.54 μg/mg insect weight). One possible explanation is that the presence of cereals and legumes as the main source of carbohydrates in Khan’s diet may have significantly increased the teneral energy reserves in the form of glucose and glycogen. The IAEA 2 diet contains yeast as an additive source of carbohydrates [25]. Khan et al. [24] stated that the addition of yeast only into the larval diet did not significantly change the overall development or male performance of mosquitoes.

Our results did not support the notion that prolongation of development time at the immature stage at a low temperature increased the energy reserves and longevity of male mosquitoes as reported by Puggioli et al. [25], Nisbet et al. [60], and Briegel & Timmermann [61]. Although high temperatures resulted in short larval developmental periods and smaller male specimens, these possessed higher energy reserves per unit weight. This was particularly true for male specimens from larvae reared at 28 °C and 32 °C, which survived significantly longer than those at 15 °C in sugar-fed experimental setup. High levels of glucose and glycogen were detected in male specimens reared at 32 °C, whereas at 15 °C, the glucose and glycogen levels were approximately 2–10-fold lower than those reared at the highest temperatures tested. Our study demonstrated that the pupae took 5–7 days to emerge to adulthood at 15 °C, whereas adulthood was reached after 1–2 days for pupae maintained at 32 °C. We speculate that energy reserves were severely depleted over a prolonged pupation period at low temperatures [62, 63], potentially accounting for the low levels of glucose and glycogen in the adult stage.

Even in the limited food condition, as much as 80% of the male population fed on Khan’s diet during the larval stage managed to survive for up to 6–8 days compared with those fed on the IAEA 2 diet that could only support male specimens for up to 6 days. In male *An. gambiae* (*s.s.*), the optimum insemination rate of mosquitoes is at 4–8 days-old [64]. For male mosquitoes, 3–7 days is thought to be an ideal age for use in an *Anopheles* SIT programme [20]. Thus, the long lifespan of released male mosquitoes may be advantageous in the SIT programme, because it increases survival of released male mosquitoes and the probability of mating with wild females in the field [23].

### Temperature tolerance

Trehalose is vital for insects to survive under extreme thermal conditions. It acts as a cryoprotectant to reduce the supercooling point of some freeze-avoiding insects by stabilizing the protein structure of the cells during desiccation and thermal stress [65]. In addition, trehalose levels are maintained in insect haemolymph during the larval stage, because it serves as a source of energy for growth, metamorphosis and chitin synthesis [18]. We observed that trehalose levels were higher, ranging from 11.33 to 18.95 µg/mg insect weight in newly emerged male specimens fed on the IAEA 2 diet during larval stages, regardless of temperature, compared with Khan’s diet, although this difference was non-significant. The high level of trehalose in male adults reared using the IAEA 2 diet increased their heat tolerance for ULT_50_ to between 40– 43 °C. In *Drosophila melanogaster* (Meigen), adults fed on protein-rich diets tended to increase their heat tolerance levels compared with those fed on carbohydrate-rich diets; the opposite applies for cold tolerance, in relation to which carbohydrate levels are more influential [66]. For Khan’s diet, the trehalose level was low at 25 °C and below (ranging from 7.53 to 8.75 μg/mg insect weight) but increased with increased temperatures; in particular, the trehalose level was above 9 µg/mg insect weight at 28 °C and 32 °C. The trehalose increment elevated upper temperature tolerance to 42 °C. However, for temperature and larval diet regimes at the lower lethal temperature, trehalose levels in male adults demonstrated no clear effects.

### Mating capacity

In this study, we observed no significant effect of diet and temperature on the percentage of inseminated female specimens. However, the ability of male specimens to inseminate more than two spermatheca capsules was temperature-dependent, in which, significantly, larger male specimens reared at 20 °C were capable of filling three spermathecae. Nevertheless, even small male specimens derived at 28 °C and 32 °C were capable of filling two spermathecae. As a result of these observations, we suggest that a smaller body may not affect male specimens’ mating capacity, whereas the findings of Ponlawat & Harrington [58] and of Armbruster & Hutchington [59] are in opposition. In SIT programs, mating competitiveness is crucial to ensure the ability of males to compete with wild males for mating. However, male mating competitiveness was not examined in the course of this experiment; this requires further study.

## Conclusions

In conclusion, larvae fed on Khan’s diet were characterized by shorter immature development time compared with those fed on the IAEA 2 diet. High temperatures of 28 °C and 32 °C elicited secondary sex ratio manipulation, causing a significant male skew for Khan’s diet. In addition, males produced under Khan’s diet at 28 °C and 32 °C had longer lifespan and superior endurance of extreme temperatures and starvation conditions, attributable to energy reserves accumulated. These characteristics may increase the mating chances of males with wild females in the field, because male survival was probably significantly improved. Specimens fed on the IAEA 2 diet exhibited higher percentage of pupation and larger males compared with those fed on Khan’s diet, but no evidence indicated reduced mating capacity among small mosquitoes fed on Khan’s diet. The mating competitiveness of male mosquitoes at various test temperatures and diet regimes is of interest, because diet regime was always size-dependent [67]. The protein levels of males fed on Khan’s diet and the IAEA 2 diet also warrant further investigation. In particular, the irradiation process may, to a certain extent, cause somatic cell damage to irradiated mosquitoes. In this respect, proteins may be vital for fitness improvement as well as mating competitiveness before release, as observed in mass-reared Mediterranean fruit fly [68].

## Supplementary information

**Additional file 1: Table S1.** The result of Studentʼs t-test for independent samples between diets (Khan’s and IAEA 2) across temperature regimes (*P* = 0.05).

## Data Availability

The data supporting the conclusions of this study are provided within the article. All datasets generated and analyzed during this study are available from the corresponding author upon reasonable request.

## Abbreviations

FAO: Food and Agriculture Organization
IAEA: International Atomic Energy Agency
LRD: laboratory rodent diet
PUFAs: polyunsaturated fatty acids
ULT: upper lethal temperature
LLT: lower lethal temperature
CV: coefficient of variance
L1–4: larval instars 1–4
