# Supplementary material for: Effects of larval diets and temperature regimes on life history traits, energy reserves and temperature tolerance of male Aedes aegypti (Diptera: Culicidae): optimizing rearing techniques for the sterile insect programmes
Source: Parasit Vectors. 2019 Dec 10;12:578. doi: 10.1186/s13071-019-3830-z (PMC6905064; doi:10.1186/s13071-019-3830-z)
Supplement: Supplementary file 1 — Additional file 1: Table S1. The result of Studentʼs t-test for independent samples between diets (Khan’s and IAEA 2) across temperature regimes (P = 0.05). [file 13071_2019_3830_MOESM1_ESM.docx]

**Additional file 1: Table S1.** The results of Independent samples *t*-test between diets (Khan’s and IAEA 2) across temperature regimes (*P* = 0.05)

| Parameters | Temp (^o^C) | t | df | Sig. (2-tailed) |
| --- | --- | --- | --- | --- |
| L1 | 15 | -7.123 | 76.871 | < 0.0001 |
|  | 20 | -5.051 | 99 | < 0.0001 |
|  | 25 | -6.215 | 62.393 | < 0.0001 |
|  | 28 | -14.214 | 57.324 | < 0.0001 |
|  | 32 | -14.764 | 98 | < 0.0001 |
| L2 | 15 | -8.941 | 92 | < 0.0001 |
|  | 20 | -4.996 | 99 | < 0.0001 |
|  | 25 | 5.871 | 96 | < 0.0001 |
|  | 28 | -1.623 | 103 | 0.108 |
|  | 32 | 2.601 | 91.114 | 0.011 |
| L3 | 15 | -8.961 | 2 | < 0.0001 |
|  | 20 | -4.364 | 75.609 | < 0.0001 |
|  | 25 | -2.251 | 89.744 | 0.027 |
|  | 28 | 2.410 | 93.923 | 0.018 |
|  | 32 | -0.812 | 98 | 0.419 |
| L4 | 15 | -5.290 | 72.311 | < 0.0001 |
|  | 20 | -13.859 | 99 | < 0.0001 |
|  | 25 | 8.376 | 96 | < 0.0001 |
|  | 28 | -3.519 | 100.222 | 0.001 |
|  | 32 | -5.193 | 98 | < 0.0001 |
| Larval stage | 15 | -10.258 | 72.409 | < 0.0001 |
|  | 20 | -16.671 | 99 | < 0.0001 |
|  | 25 | 5.249 | 96 | < 0.0001 |
|  | 28 | -8.362 | 103 | < 0.0001 |
|  | 32 | -8.750 | 72.762 | < 0.0001 |
| Pupal stage | 15 | 3.032 | 31.550 | 0.005 |
|  | 20 | -6.336 | 88 | < 0.0001 |
|  | 25 | 3.405 | 62 | 0.001 |
|  | 28 | -0.943 | 73 | 0.349 |
|  | 32 | 0.404 | 50.548 | 0.688 |
| Total immature | 15 | -5.153 | 45 | < 0.0001 |
|  | 20 | -13.987 | 88 | < 0.0001 |
|  | 25 | 4.250 | 62 | < 0.0001 |
|  | 28 | -7.801 | 51.129 | < 0.0001 |
|  | 32 | -12.552 | 65.641 | < 0.0001 |
| Pupation (%) | 15 | 0.535 | 3.382 | 0.626 |
|  | 20 | 0.363 | 6 | 0.729 |
|  | 25 | 0.009 | 6 | 0.993 |
|  | 28 | -3.467 | 6 | 0.013 |
|  | 32 | -4.620 | 6 | 0.004 |
| Adult eclosion (%) | 15 | -0.418 | 6 | 0.691 |
|  | 20 | 1.000 | 3 | 0.391 |
|  | 25 | -1.622 | 3 | 0.203 |
|  | 28 | 2.825 | 6 | 0.030 |
|  | 32 | 1.670 | 6 | 0.146 |
| Cephalothorax length | 15 | 0.673 | 38 | 0.505 |
|  | 20 | 5.439 | 28 | < 0.0001 |
|  | 25 | 1.689 | 33 | 0.101 |
|  | 28 | -1.302 | 29 | 0.203 |
|  | 32 | 1.350 | 29 | 0.188 |
| Wing length | 15 | 0.027 | 37 | 0.978 |
|  | 20 | -1.586 | 40 | 0.121 |
|  | 25 | 1.782 | 34 | 0.084 |
|  | 28 | -3.130 | 39 | 0.003 |
|  | 32 | -6.446 | 39 | < 0.0001 |
| Adult longevity | 15 | 13.758 | 71.857 | < 0.0001 |
| (water-fed) | 20 | 12.004 | 128 | < 0.0001 |
|  | 25 | 8.585 | 127 | < 0.0001 |
|  | 28 | 18.002 | 121 | < 0.0001 |
|  | 32 | 13.990 | 88.338 | < 0.0001 |
| Adult longevity | 15 | 1.883 | 28 | 0.070 |
| (sugar-fed) | 20 | 2.872 | 48 | 0.006 |
|  | 25 | 1.124 | 42 | 0.267 |
|  | 28 | 4.818 | 16.579 | < 0.0001 |
|  | 32 | 1.784 | 34 | 0.083 |
| Glucose | 15 | 2.445 | 11 | 0.033 |
|  | 20 | 0.797 | 11 | 0.442 |
|  | 25 | 0.088 | 6 | 0.933 |
|  | 28 | 0.896 | 6 | 0.405 |
|  | 32 | 3.885 | 7 | 0.006 |
| Glycogen | 15 | 3.126 | 11 | 0.010 |
|  | 20 | 0.948 | 11 | 0.363 |
|  | 25 | -3.837 | 2.384 | 0.046 |
|  | 28 | 1.924 | 6 | 0.103 |
|  | 32 | 3.634 | 7 | 0.008 |
| Trehalose | 15 | -2.222 | 11 | 0.048 |
|  | 20 | -3.133 | 4.812 | 0.027 |
|  | 25 | -2.537 | 6 | 0.044 |
|  | 28 | -1.400 | 6 | 0.211 |
|  | 32 | -0.667 | 7 | 0.526 |
| Female insemination | 15 | -1.810 | 6 | 0.120 |
|  | 20 | -0.515 | 6 | 0.625 |
|  | 25 | -0.230 | 6 | 0.825 |
|  | 28 | -0.322 | 6 | 0.758 |
|  | 32 | -0.140 | 6 | 0.989 |
|  |  |  |  |  |
